# Trust in Humanitarian Aid From the Earthquake in 2017 to COVID-19 in Iran: A Policy Analysis

**DOI:** 10.1017/dmp.2020.54

**Published:** 2020-03-27

**Authors:** Hamed Seddighi

**Affiliations:** Student Research Committee, University of Social Welfare and Rehabilitation Sciences, Tehran, Iran

**Keywords:** COVID-19, disasters, humanitarian aid, trust

## Abstract

The earthquake of November 2017, the great flood of April 2019, and the COVID-19 outbreak in 2020 are 3 major emergencies in Iran during the last 3 years. A common issue in all of these crises seems to be the issue of “trust.” Official authorities, including the Iranian President, ministers, and the judiciary system, tried to gain people’s trust by either changing policies or developing new ones. In August 2019, the new law on crisis management in Iran went into effect and the issue of public donation has been considered, too. Also, in their response to the COVID-19 outbreak, Iranian officials ordered all sectors to cooperate with the Ministry of Health and provide it with all necessary facilities. Therefore, it seems that new policies are still needed to overcome mistrust in Iran at times of emergency. Developing a policy on donation management was the first step, and there are several factors that could have contributed to the perception of the mistrust and failure in emergency missions. Mistrust can be the result of different causes, including but not limited to lack of knowledge on capabilities and efficiencies of humanitarian organizations, engagement of a wide range of organizations from different categories, extension of mistrust of an organization to other emergency organizations in the area or all of operation, lack of unity in emergency response, and poor public relations.

An earthquake with a moment magnitude of 7.3 occurred on November 12, 2017, near the Iran–Iraq border, Kermanshah Province in Iran. At least 630 people were killed and more than 8100 were injured, as well as much more unaccounted for. It was the deadliest earthquake of 2017.^1^ In another disaster in Iran, from mid-March to April 2019, during Iran New Year’s holidays (Nowruz), widespread flooding and flash flooding affected at least 26 of Iran’s 31 provinces. The situation in 4 provinces was worse, including Golestan, Fars, Khuzestan, and Lorestan. More than 70 people were killed and 260 000 displaced. Many people were traveling for the Nowruz holiday and were injured or killed when their cars were swept off of roads, especially in Fars province flooding.^2^ Further, according to Red Crescent, 2 million people were in need of humanitarian aid due to the devastating floods.^3^ During the 2019–2020 coronavirus (COVID-19) outbreak, Iran has become 1 of the top 4 countries reporting COVID-19 deaths and infected patients.^4^ All provinces in Iran have reported confirmed COVID-19 cases.

## SCOPE OF THE PROBLEM

After the earthquake in 2017, many international and national organizations, governments, celebrities, and Iranian citizens helped disaster victims with various forms of humanitarian aid: cash, shelter, and food.^2^ Since the early days of the earthquake, many people had challenged the integrity of aid distributions.^2,5-8^ They discussed humanitarian aid and their donations in popular online social media and newspapers in Iran.^6,8^ This time, more people preferred to donate to disaster victims through celebrities and influencers rather than official organizations. A reason could be donators’ declining trust in the government and official organizations.^6,8^ Iranian newspapers published articles discussing the multilateral mistrust between the government and the public, including celebrities; celebrities and the government; humanitarian organizations and celebrities; and nongovernmental organizations (NGOs) and the like.^2,6,8^ It indicates serious interpersonal and interorganizational mistrust. Government and humanitarian organizations, such as Red Crescent, with many humanitarian activities did not report to people about their activities in detail. The Iranian Parliament, a special committee for Kermanshah earthquake, mentioned some shortcomings in their report.^9^ Their report emphasized that the coordination among different organizations in the area was poor, humanitarian organizations were not prepared for the earthquake logistically, and also those organizations failed in aid distribution to the affected people.^9^ The report confirmed people’s mistrust in the humanitarian organizations and claimed that the main reason was the lack of control relating to online social media.^9^

Since the earthquake in 2017, the issue of trust has been obvious in every emergency, including the flood in 2019 and, finally, the COVID-19 outbreak in 2019–2020. During the outbreak, Iranian authorities spoke clearly about people’s mistrust of the government. They asked people to trust the services provided by the government. The Iranian President, during 2 speeches in 10 days, emphasized the issue of trust. “Trust is a principle for us and truth should be told to people,” the president stated in the national prevention of and response to the COVID-19 headquarters meeting on February 25, 2020.^10^ He repeated these words 10 days later in the cabinet meeting and asked people to trust in health systems statements and to stay at home.^11^ Also, the spokesperson of the Iranian Government in the early days of the outbreak announced that “the most important treatment for coronavirus is the public’s trust in the health system and government.”^12^ The top officials’ speeches about trust have revealed that there is a problem. They knew the mistrust would negatively influence the COVID-19 outbreak response. It is obvious in the words of the public, media, and some authorities that there is something wrong with the public trust. “Late and incomplete informing activities on coronavirus have even further weakened the social trust already damaged heavily during the events of the past months,” stated the chairman of the City Council of Tehran in the meeting of the council.^13^

## POLICY WINDOW FOR GOVERNMENT

Response and recovery operations following the earthquake in 2017 showed several challenges, including inconsistency between the government and the public; lack of unity in response, public relations, and logistics; and failure to fulfill commitments.^2,6,8,9^ These challenges were reflected in official speeches and reports.^9^

After the earthquake in 2017 and during the flood in 2019, the Iranian judiciary banned any aid collection by celebrities in disasters, and blocked their more than 100 bank accounts used for collecting humanitarian aid.^14,15^ That was a decision previously proposed by the Iranian Parliament’s special committee for the Kermanshah earthquake.^9^ According to that judicial order, the Red Crescent and Imam Khomeini Relief Foundation are 2 sole organizations exclusively in charge of collecting humanitarian aid from inside and outside of Iran.^6^ Iranian’s attorney general declared that any NGO intending to participate in such process must ask 1 of these 2 organizations for permission.^15^

In August 2019, a new law on crisis management in Iran was announced in which the problem of public donation in disasters has been mentioned.^16^ The new law emphasized the following:

*Iranian Red Crescent Society in cooperation with […] shall take the necessary measures to attract, direct and distribute domestic and foreign nongovernmental assistance according to Article 80 of the Constitution. Any interference with, seizure, use, and possession of such assistance by natural or juridical persons is prohibited by law and is considered a criminal act of unlawful possession of public or state property realized as committing an offense under Islamic Penal Code.* ^16^

The above law has reduced the debates and concerns about the unity in humanitarian aid.

Different spokespersons and lack of unity in command systems are still 2 major problems in emergencies, which enhance mistrust in Iranian society. In the COVID-19 outbreak, the government tried to solve these problems by applying new policies. Iranian supreme leader stated in a speech on COVID-19, “*All sectors in the country should cooperate and provide the necessary facilities to the Ministry of Health, which is at the forefront of the fight against the disease… the armed forces and entities connected with the Office of the Leader are also obliged to do the same… our officials have been informing the public since the first day honestly and transparently.”* ^17^

Regarding the order of the Iranian supreme leader, COVID-19 appears to be a unique case in emergency response in terms of unity of spokespersons, unity of command systems (Ministry of Health), and an order necessitating all organizations to fully cooperate with the Ministry of Health.

## DISCUSSION

The so-called “What’s the problem represented to be?” is an approach to policy analysis by Carol Bacchi.^18^ In her approach to policy analysis, she addresses 6 questions about the problem, including the (1) problem itself, (2) presupposition for representation of the problem, (3) problem’s origin, (4) policy silences, (5) effect produced by the representations of the problem, and (6) unproblematic issues.^18^ This paper focuses on the sixth question, “What is left unproblematic in this problem representation?”

The Iranian Government realized there was a problem with humanitarian aid and employed a legislative remedy. But mistrust remains. For example, in October 2019, people in a small town in Iran had a mass protest, after infection of a huge number of people to HIV.^19^ They protested because they thought the virus came from a local health care vaccination center. It seems that they lacked enough knowledge about the cause of their infection and even the explanation by the Ministry of Health could not persuade them and they continued their protest.^19^ In fall of 2019, there was mistrust of the influenza vaccination, and many people refused to use it. After a wave of influenza epidemic in November 2019, the Ministry of Health blamed the people for their refusal to use the vaccination. Meanwhile, the head of the Centre for Communicable Diseases Management at the Iranian Ministry of Health said: “Despite the fact that some people are campaigning against the vaccine, the vaccine produced in our country is the best type of influenza vaccine.”^20^ In the great flood in 2019, many people in different towns and provinces refused to evacuate after evacuation orders, and governors mentioned that as a great challenge for response operation.^21^

In a disaster, different stakeholders have roles in humanitarian aid. These stakeholders forming a network have certain characteristics: their network is formed rapidly, they come from different contexts (organizations, cultures, provinces, or countries), they have a shared space for conversation and coordination, and they carry out an urgent grave mission.^22^ These stakeholders or humanitarian organizations should coordinate with each other for a better response, while simultaneously humanitarian organizations compete with each other.^22^ They compete for both the resources (financial or material from donors) and media attention. This competition in humanitarian aid may lead to overlapping duties or even worse – failure to deliver aid to the disaster-affected people.^22^ This is what happened in the humanitarian response for the disaster victims in the Kermanshah earthquake in 2017 and great flood in 2019. In spite of Iran’s new law on crisis management emphasizing the necessity of allocation of cash and kind donation to Red Crescent Society, the problems are still unsolved.

According to Tatham and Kovács,^22^ trust in humanitarian aid in disasters depends on some elements, including (1) Third party information: enough information on professional reputation and skills of different players in a disaster relief network will reduce the risk of inefficiency of another party; (2) Rules: including humanitarian response procedures will protect the operation from interpersonal and interorganizational mistrust; (3) Category: players involved in disaster relief come from different categories of gender, race, ethnicity, religion, age, and mission – that is, army and Red Cross; as a result of such differences, more strong communications and relationships will be established among them and it will remove mistrust; and (4) Role: role-based trust or competence trust is formed based on 1 party’s trust in the other party’s competence because of its skills, knowledge, and good history.

So, it seems that new policies are still needed for overcoming interpersonal and interorganizational mistrust in Iran at the times of emergency. Developing a policy on donation management was the first step, and there are many factors that could have contributed to the perception of mistrust and the reasons of failure in emergency missions. Mistrust can be the result of different causes, including lack of knowledge about capabilities and efficiencies of humanitarian organizations, involvement of organizations from different categories (Red Crescent Society, Army, Welfare Organization, Crisis Management Organization, NGOs, celebrities, clergies, and the like), extension of mistrust of an organization to other emergency organizations in the area or all of operation, lack of unity in emergency response, and poor public relations.

## IMPLICATIONS FOR PRACTICES AND POLICIES

- Modifying the system of need assessment in disasters can be a great step for creating trust between people and organizations.
- Dialogue between humanitarian organizations, celebrities, and people before disasters and in the preparedness phase will result in a better response during and after disasters.
- Improving report mechanisms by all actors involved in disaster relief can help improve social capital and trust.
- Participatory humanitarian logistics from planning to distribution in different phases like preparedness, response, and recovery is 1 way to enhance transparency and trust.
- Humanitarian organizations and other actors in disasters should get help from famous people and influencers to send their message to people on social media.
- Discussing information about other actors in disaster and dispatching trained individuals to respond is crucial for making interpersonal and interorganizational trust.
- Modifying and formulating policies, rules, and regulations for building trust at all levels and enhancing coordination between actors are vital.
- Disaster response by actors from different categories (ie, police, army, NGOs, celebrities, and the like) need a communication atmosphere before and during disasters, and it should start from the first phases of disaster risk management, such as mitigation and preparedness.
- Professional training for humanitarian logisticians organizationally and inter-organizationally is needed.
